# OA07 Pregnancy in a patient with obstetric antiphospholipid syndrome complicated by subchorionic haematoma and cervical shortening

**DOI:** 10.1093/rap/rkag113.007

**Published:** 2026-11-03

**Authors:** Julia Graszkiewicz, Lauren Dolan

**Affiliations:** South Warwickshire NHS Foundation Trust, Warwick, United Kingdom; South Warwickshire NHS Foundation Trust, Warwick, United Kingdom

## Abstract

**Introduction:**

Obstetric antiphospholipid syndrome (OAPS) is an autoimmune disorder characterised by recurrent pregnancy complications, with persistent positivity for antiphospholipid antibodies. Adverse pregnancy outcomes associated with OAPS include recurrent miscarriage, pre-eclampsia, intrauterine growth restriction (IUGR), prematurity and stillbirth. Without intervention, only 25% of APS patients successfully deliver a healthy neonate, compared to 70% with treatment. The case presented is that of a patient diagnosed with OAPS under Haematology care who, despite additional complications, was able to deliver healthy twins after in vitro fertilisation (IVF) conception; thus demonstrating the importance of early diagnosis and careful planning of obstetric care.

**Case description:**

A woman in her 20s with a history of polycystic ovary syndrome (PCOS) and subfertility developed livedo reticularis during her first pregnancy, conceived via in vitro fertilisation (IVF). This singleton pregnancy ended in stillbirth at 23 weeks, complicated by early retroplacental haematoma, severe preterm prelabour rupture of membranes (PPROM), and placental abruption. Borderline elevated anticardiolipin antibodies, together with livedo reticularis and severe obstetric complications, led to a diagnosis of obstetric antiphospholipid syndrome (OAPS), which was later supported by strongly positive anti-β2-glycoprotein-1 antibodies (>100). Aspirin therapy was continued due to persistent skin mottling and paraesthesia.

The patient’s care remained under haematology with Rheumatology assessment finding no evidence of coexisting connective tissue disease with negative antinuclear antibodies, rheumatoid factor, dsDNA antibodies, complement levels, immunoglobulins, and erythrocyte sedimentation rate.

Two years later, following a successful IVF cycle resulting in a monochorionic diamniotic twin pregnancy, the patient was managed with low-dose aspirin and low-molecular-weight heparin (LMWH). Anticoagulation was suspended because of a subchorionic haematoma detected at 5 weeks’ gestation. Hydroxychloroquine was initiated but discontinued at 20 weeks because of adverse effects. Following resolution of the haematoma, aspirin and enoxaparin were restarted.

At 22 weeks, cervical shortening was identified, indicating an increased risk of spontaneous preterm birth. Management included oral progesterone, strict bed rest, and close foetal growth surveillance. The patient subsequently delivered healthy twins by Caesarean section at 33 weeks, with no evidence of intrauterine growth restriction. Enoxaparin was continued for six weeks postpartum.

Following delivery, she developed inflammatory arthralgia affecting the small joints of the hands and feet. Ultrasound confirmed low-grade synovitis, leading to a diagnosis of seronegative rheumatoid arthritis. Treatment with methotrexate resulted in good symptomatic control.

**Discussion:**

The pathophysiology of OAPS is complex and affects all stages of pregnancy. Common histopathological features include placental infarction, impaired spiral artery remodelling and decidual inflammation, providing an explanation for the commonest manifestations of OAPS, such as pre-eclampsia and placental insufficiency. The effect of antibodies on uterine decidualisation and implantation may contribute to recurrent miscarriage and primary infertility.

Current evidence supports the combined use of low-dose aspirin and LMWH to reduce miscarriage rates. Immune-modulating therapies such as hydroxychloroquine (HCQ), which reduces placental inflammation and aids trophoblast function, have been recommended as an adjunct in refractory cases. Close surveillance with foetal scans and umbilical and uterine Doppler sonography is recommended.

In our case the management of APS was complicated by the development of a subchorionic haematoma (SCH). Due to concerns over bleeding risk, aspirin and LMWH were paused. HCQ provided an alternative when standard therapy was not suitable.

The pathogenesis of SCH is uncertain but has been linked with the presence of autoantibodies and use of assisted reproduction technology. However, the exact impact of SCH on pregnancy outcomes remains unclear. As combined anticoagulation therapy is a mainstay of treatment in OAPS, the risks of stopping or continuing treatment should be carefully weighed up. Further research is paramount to understand the significance of SCH and how it should be managed in OAPS.

It is well-recognised that patients with autoimmune diseases such as rheumatoid arthritis see improvement in disease activity during pregnancy with postpartum flare, due to the immunomodulatory effect of pregnancy. This protective effect can last into the first 3 months post-partum. The development of an inflammatory arthritis within 14 weeks of birth, as seen in this case, is unusual. Clinicians must be aware of the association between autoimmune disease and antiphospholipid antibodies, and carefully evaluate patients for features of systemic inflammatory disorders.

**Key learning points:**

• This case was complex due to multiple independent risk factors for adverse pregnancy outcomes, including obstetric antiphospholipid syndrome (OAPS), multiple gestation, and conception through assisted reproductive technology (ART). Further research is needed to better understand the interaction between these factors and their combined impact on maternal and foetal outcomes, particularly when existing literature focuses largely on singleton pregnancies.

• This patient displayed typical clinical and laboratory findings of OAPS, including livedo reticularis and late obstetric complications. Anti-cardiolipin antibody was below the threshold described by the Sapporo criteria, however a strongly positive beta-2-glycoprotein-1 antibody confirmed the diagnosis. Early recognition of APS enabled timely initiation of prophylactic therapy and close antenatal surveillance, contributing to a successful second pregnancy. This underlines the importance of screening patients with clinical features suggestive of APS to facilitate early intervention and optimise pregnancy outcomes.

• Management of OAPS should be individualised, taking into account maternal and foetal factors as well as changing clinical circumstances. In this case, combined anticoagulation and hydroxychloroquine were used, with treatment temporarily modified when bleeding risk associated with subchorionic haematoma (SCH) and maternal side effects outweighed the benefits.

• Both OAPS and ART have been associated with an increased risk of SCH, although its effect on pregnancy outcomes remains uncertain, it has been postulated that SCH is associated with more severe coagulopathy, immune disorder and increased risk of preterm delivery. Further studies are required to clarify the clinical significance and guide management decisions.

• Finally, this patient developed seronegative inflammatory arthritis postpartum, highlighting the increased risk of subsequent autoimmune disease in patients with OAPS. Ongoing clinical assessment for rheumatological disorders, together with appropriate counselling and safety-netting, may facilitate earlier recognition and management of emerging autoimmune disease.

